# Elucidating Pancreatic Ductal Adenocarcinoma Carcinogenesis at Single-Cell Resolution and Identifying Subtype Specific Drug Candidates

**DOI:** 10.3390/ijms262412031

**Published:** 2025-12-14

**Authors:** Jing Chen, Hui Jiang, Hui Chen, Kuan Yang, Kaiyue Yang, Mingyao Sun, Na Lv, Bolin Ren, Xinyi Lin, Xia Li, Yunpeng Zhang, Congxue Hu

**Affiliations:** College of Bioinformatics Science and Technology, Harbin Medical University, Harbin 150081, China; chenjing1208@hrbmu.edu.cn (J.C.); jianghui152001@163.com (H.J.); 2024020526@hrbmu.edu.cn (H.C.); yangkuan@hrbmu.edu.cn (K.Y.); yangkaiyue0904@hrbmu.edu.cn (K.Y.); 18547600199@163.com (M.S.); 15944936801@163.com (N.L.); qq3389973206@163.com (B.R.); linxinyi_lxy@163.com (X.L.); lixia@hrbmu.edu.cn (X.L.)

**Keywords:** scRNA-seq, PDAC, preneoplastic stage, carcinogenesis, drug candidate

## Abstract

Although single-cell analyses have advanced our understanding of pancreatic ductal adenocarcinoma (PDAC), most studies to date have focused on primary and metastatic tumors. Here, we map cell composition, phenotypic plasticity, and microenvironmental remodeling from human normal pancreas through preneoplastic lesions to PDAC, with the preneoplastic phase recognized as a critical window for carcinogenesis. We pinpoint genes that are persistently dysregulated throughout malignant transformation and are associated with a poor prognosis. Focusing on ductal and acinar cells as the principal origins of PDAC, we delineate malignant preneoplastic cell clusters that exhibit strong carcinogenic potential. Immune profiling reveals marked expansion and functional reprogramming of macrophages during disease progression. Integrative analysis with human PDAC bulk transcriptomic cohorts identifies candidate compounds, such as Brefeldin A, with potential for intervention in preneoplastic disease. Together, our study elucidates dynamic molecular and cellular mechanisms underlying PDAC carcinogenesis and provides actionable insights for early intervention and targeted therapy.

## 1. Introduction

Pancreatic ductal adenocarcinoma (PDAC) is one of the most lethal malignancies worldwide. Epidemiological studies indicate that the five-year survival rate for patients diagnosed at an advanced stage is only 5–10% in most regions [1]. In contrast, early detection and active treatment can effectively improve patient prognosis. Retrospective analyses show that approximately 80% of patients with stage IA or IB PDAC achieve five-year survival rates up to 50%, whereas those with advanced disease rarely exceed 10% [2]. The carcinogenesis of PDAC typically arises through a multistep series of precursor lesions, with a prolonged timeline for progression to invasive neoplasia [3], providing a valuable window for early intervention. Therefore, intervening at the preneoplastic stage may effectively prevent progression to malignancy and ultimately improve overall patient outcomes.

Clinical management of pancreatic ductal adenocarcinoma (PDAC) remains challenging. Surgical resection is the only potentially curative option, yet most patients are unresectable at diagnosis [4]. Systemic chemotherapy—primarily gemcitabine-based regimens or FOLFIRINOX—remains the standard of care [5], but efficacy is limited by intrinsic or acquired resistance. Consequently, the focus has shifted to targeted approaches, including modulating the tumor microenvironment or reversing resistance (e.g., by regulating drug transporters such as hENT1 to enhance chemosensitivity [6]) and inhibiting oncogenic kinases (e.g., KRASG12C) [7]. Novel pan-RAS inhibitors (e.g., ADT-1004) show preclinical potential to overcome diverse KRAS mutations and resistance mechanisms [8]. Thus, elucidating the cellular and molecular pathogenesis of PDAC is imperative for developing novel interventions.

Single-cell RNA sequencing (scRNA-seq) has provided unprecedented resolution for dissecting the cellular and transcriptional heterogeneity of tumors and their microenvironment [9]. In PDAC, scRNA-seq has revealed complex cellular states and their dynamic transitions, elucidating the molecular mechanisms of malignant progression [10,11]. However, current studies predominantly focus on primary and metastatic tumors. Therefore, systematically establishing a single-cell atlas that delineates the evolutionary process from normal pancreas through preneoplastic lesions to PDAC is of significant scientific value for elucidating the mechanisms underlying PDAC carcinogenesis.

The cellular origin of PDAC has long been a subject of debate. Traditionally, its characteristic ductal morphology has led to the assumption that malignant transformation originates from ductal cells [12]. However, recent studies employing genetically engineered mouse models have revealed remarkable plasticity in acinar cells, demonstrating that acinar-to-ductal metaplasia (ADM) can initiate pancreatic intraepithelial neoplasia (PanIN), the principal precursor lesion of PDAC [13]. Beyond the cellular origin, increasing evidence suggests that immune–epithelial interactions within the tumor microenvironment and macrophage-driven tissue remodeling are also important drivers of PDAC progression [14,15].

In this study, we systematically analyzed single-cell data from normal, preneoplastic, and PDAC samples, comprehensively delineating the dynamic changes in the microenvironment during PDAC carcinogenesis. We identified key malignant subclusters in the preneoplastic stage that drive disease progression and mapped their interactions with immune cells. Furthermore, by integrating single-cell features into the bulk transcriptomic level and leveraging LINCS data, we pinpointed potential compounds capable of intervening in preneoplastic lesions.

## 2. Results

### 2.1. Dynamic Landscape Uncovers Persistently Dysregulated Genes Throughout PDAC Carcinogenesis

**Figure 1:**
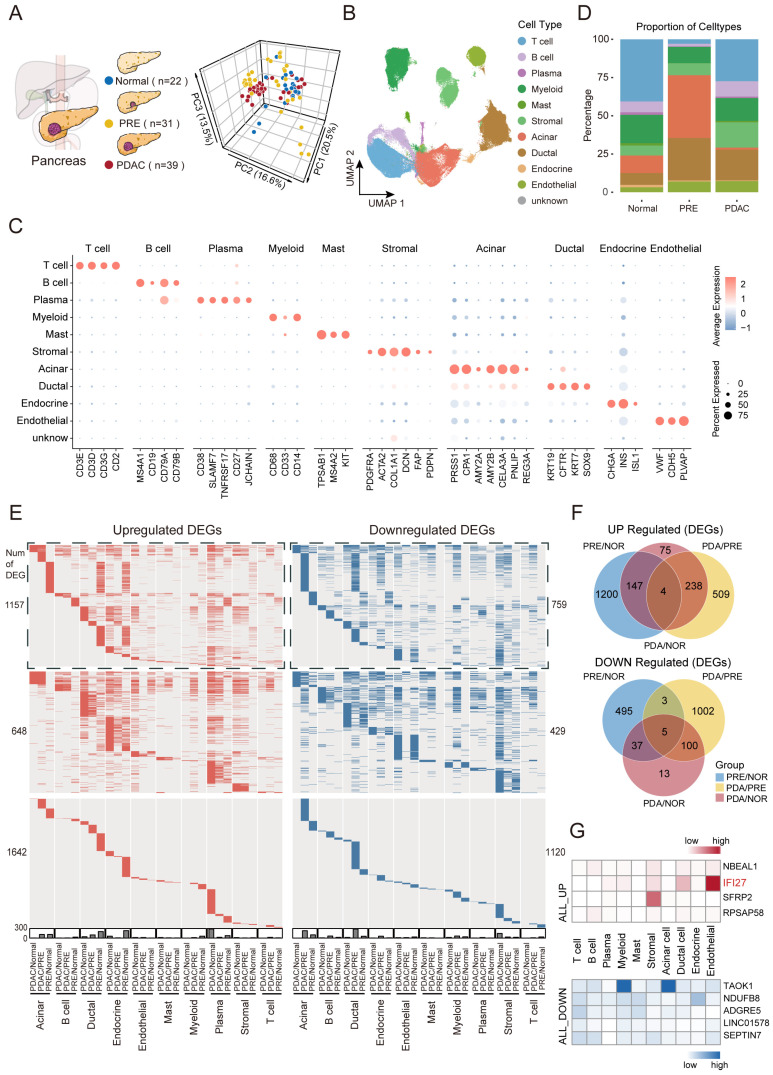
Single-cell transcriptomic landscape and gene expression dynamics. (**A**) **Left**: Overview of pancreatic progression from Normal to PRE and PDAC, with single-cell sample numbers (Normal: 22, PRE: 31, PDAC: 39). **Right**: Three-dimensional PCA of sample gene expression by stage (Normal, PRE, PDAC). Distances between dots reflect differences between samples. (**B**) UMAP visualization of 170,159 single cells, colored by cell type. (**C**) Dot plot of selected marker gene expression across major cell types. (**D**) Cell type proportions in Normal, PRE, and PDAC stages shown as stacked bar plots. (**E**) Heatmaps of scRNA-seq data showing upregulated (**left**, red) and downregulated (**right**, blue) differentially expressed genes (DEGs) by cell type for PRE vs. NOR (PRE/NOR), PDA vs. PRE (PDA/PRE), and PDA vs. NOR (PDA/NOR) comparisons. Genes without significant expression changes are indicated in gray. DEGs shared by at least two cell types are highlighted at the top, those shared by at least two comparisons are shown in the middle, and DEGs unique to each cell type are displayed at the bottom. The number of genes is indicated. Gene names for these groups are listed in Supplementary Table S2 in the same order. (**F**) Venn diagrams showing the overlap of upregulated (**top**) and downregulated (**bottom**) DEGs among the three comparisons, as listed in Supplementary Table S3. (**G**) Expression heatmaps depicting genes persistently upregulated (**top**) or downregulated (**bottom**) across cell types, defined by the intersection of three pairwise Venn comparisons.

To delineate the dynamic changes in the microenvironment throughout carcinogenesis, we integrated single-cell samples spanning 22 normal pancreas (Normal), 31 preneoplastic lesions (PRE), and 39 pancreatic ductal adenocarcinomas (PDAC), all of which are publicly available (Figure 1A Left; Supplementary Table S1). Three-dimensional principal component analysis (PCA) revealed that PRE and PDAC samples were closely positioned in transcriptomic space, indicating a high degree of molecular similarity between these two stages (Figure 1A Right). After stringent quality control, normalization, batch-effect correction, and dimensionality reduction (Supplementary Figure S1A), we obtained 170,159 high-quality cells, comprising 34,676 (20.4%) from the Normal stage, 66,792 (39.2%) from the PRE stage, and 68,691 (40.4%) from the PDAC stage (Supplementary Figure S1B). Leveraging canonical marker genes, we annotated ten major cell types, including T cells, B cells, plasma cells, myeloid cells, mast cells, stromal cells, acinar cells, ductal cells, endocrine cells, and endothelial cells (Figure 1B,C; Supplementary Figure S1C).

We further investigated the dynamic remodeling of the cellular microenvironment during carcinogenesis (Figure 1D). Immune cells were predominant in the Normal stage. However, their proportion sharply declined in the PRE stage and then rebounded in the PDAC stage (69.5%, 15.7%, and 54.3%, respectively). Conversely, ductal and acinar cells markedly expanded from Normal to PRE, with their fractions subsequently contracting in PDAC (19.2%, 68.6%, and 21.4%, respectively) (Supplementary Figure S1D). Consistent with prior single-cell analyses of PDAC across clinical stages [16], our results reveal dynamic shifts in cellular composition from PRE to PDAC, characterized by increasing immune cell infiltration and decreasing ductal cell abundance. Importantly, our findings not only corroborate the transition from PRE to PDAC but also extend these insights by delineating microenvironmental evolution from Normal to PRE.

To systematically characterize dynamic transcriptomic alterations during PDAC carcinogenesis at the single-cell level, we performed differential expression analyses across major cell types and disease stages (Figure 1E; Supplementary Table S2). By quantifying differentially expressed genes (DEGs) for each cell type between PDAC-PRE and PRE-Normal stages, we found a marked increase in DEG numbers during tumor progression in all cell types except ductal, endocrine, and endothelial cells (Supplementary Figure S1E). Specifically, we identified 1351 upregulated and 541 downregulated DEGs between PRE and Normal (PRE/NOR), 752 upregulated and 1110 downregulated DEGs between PDAC and PRE (PDA/PRE), and 465 upregulated and 156 downregulated DEGs between PDAC and Normal (PDA/NOR) (Figure 1F; Supplementary Table S3). Notably, 52.3% of upregulated and 67.3% of downregulated DEGs in the PDA/NOR comparison overlapped with those in the PDA/PRE group, indicating persistent gene activation or repression throughout carcinogenesis.

We defined aberrant genes as those consistently differentially expressed across all comparisons, reflecting sustained upregulation or downregulation during disease progression (Figure 1G). Further analysis revealed that *IFI27*, a persistently upregulated gene, was primarily expressed in endothelial and ductal cells. *IFI27* is among the most upregulated genes in infection, cancer, inflammation, and autoimmune disorders [17], and its modulation of immune and metabolic pathways in the tumor microenvironment promotes PDAC progression and is associated with poor prognosis [18]. In contrast, *TAOK1* is persistently downregulated and is primarily expressed in myeloid and acinar cells. As a negative regulator of *IL-17*-mediated signaling and inflammation [19], *TAOK1* shows markedly reduced expression in the colonic tissues of patients with ulcerative colitis and colorectal adenocarcinoma [20].

In summary, our findings reveal the dynamic remodeling of the microenvironment and persistent gene dysregulation throughout PDAC carcinogenesis.

### 2.2. Identification and Characterization of Malignant Preneoplastic Clusters in Ductal and Acinar Cells

**Figure 2:**
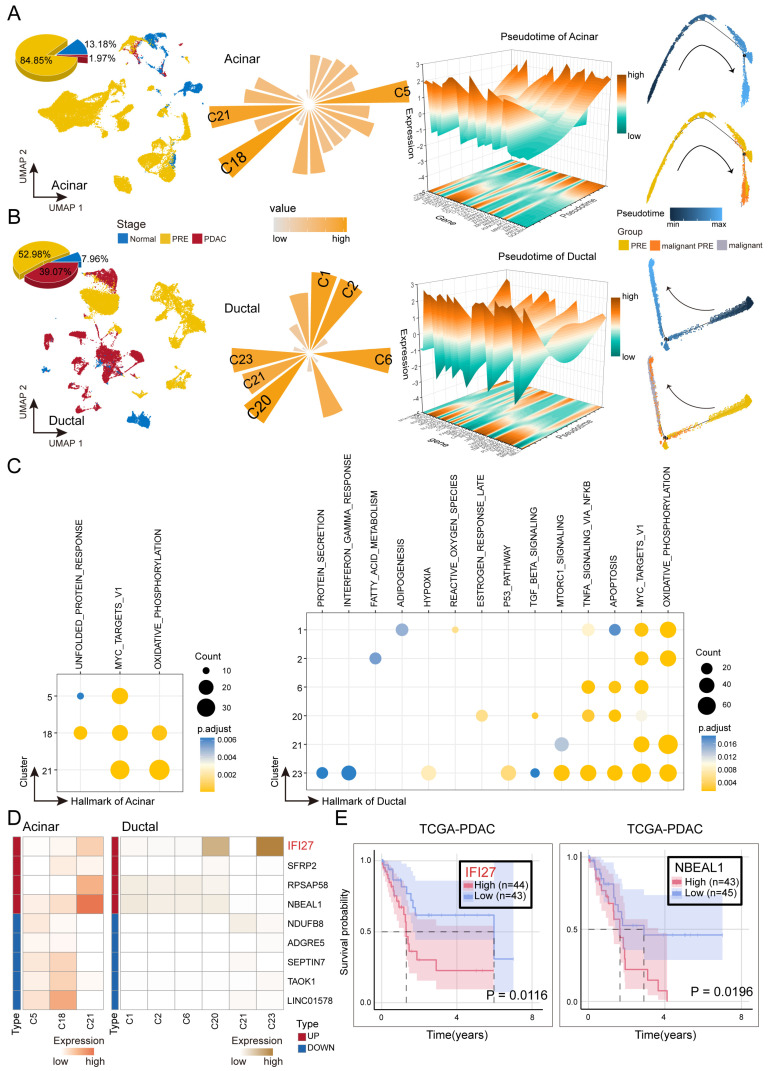
Identification and Characterization of Malignant Preneoplastic Subclusters in Ductal and Acinar Cells. (**A**) UMAP projections show the distribution of acinar cells across stages, with 3D pie charts summarizing composition. Rose plots display correlations between PRE clusters and inferCNV-identified malignant cells. Pseudotime heatmaps and trajectories illustrate transitions from PRE, through malignant PRE, to PDAC. Arrowheads on the trajectories denote the inferred directionality of pseudotemporal progression. (**B**) As in (**A**), for ductal cells. (**C**) Bubble plots of Hallmark pathway enrichment in potentially malignant pre-malignant acinar (**left**) and ductal (**right**) subclusters. Bubble size indicates gene count; color reflects adjusted *p* value. (**D**) Heatmaps of persistently dysregulated gene expression in potentially malignant PRE clusters of acinar (**left**) and ductal (**right**) cells, grouped by regulation direction (upregulated, top; downregulated, bottom). (**E**) Survival analysis of genes at the intersection of trajectory-associated and persistently dysregulated genes. Two representative genes identified from TCGA PDAC datasets are significantly associated with poor patient prognosis (highly significant *p* values).

Given the ongoing uncertainty regarding the cellular origin of PDAC, we examined ductal and acinar cells as putative malignant progenitors and characterized their single-cell dynamics throughout carcinogenesis (Figure 2A,B).

We isolated ductal and acinar cell populations and systematically quantified their distributions across different disease stages. Within the ductal cells, 52.98% of cells originated from the PRE stage and 39.07% from PDAC. In contrast, the majority of acinar cells originated from the PRE stage (84.85%), with a minimal fraction from PDAC (1.97%). When assessing their proportions within the entire cell population, ductal and acinar cells exhibited similar abundance in the Normal stage. Acinar cells predominated in PRE but were substantially reduced in PDAC, where ductal cells became the dominant population (Supplementary Figure S1D). These dynamic shifts suggest that acinar cells undergo acinar-to-ductal metaplasia (ADM) toward a ductal phenotype, representing not only an adaptive response to inflammation and microenvironmental stress during carcinogenesis, but also providing the cellular and architectural foundation for the development of PDAC. The resultant ductal-like cells exhibit heightened sensitivity to oncogenic signals, thereby facilitating tumor initiation and progression [21,22,23].

Given the critical role of preneoplastic lesions as intermediates in malignant transformation, we aimed to identify preneoplastic subclusters with carcinogenic potential. Using B cells as a reference, we applied inferCNV analysis to distinguish malignant cells within PDAC stages (Supplementary Figure S2A,B). We then assessed Pearson correlations between PRE subclusters and inferCNV-defined malignant clusters. PRE subclusters exhibiting strong correlations (r > 0.75) were designated as malignant PRE clusters (Supplementary Figure S2C; Supplementary Table S4). To validate the biological continuity of these subclusters, trajectory inference with Monocle2 revealed a continuous progression from normal PRE cells through malignant PRE cells to malignant cells, as depicted by trajectory and pseudotime heatmaps.

To investigate the molecular mechanisms of malignant PRE subclusters, HALLMARK pathway enrichment analysis was conducted. Both ductal and acinar subclusters were significantly enriched in the OXIDATIVE_PHOSPHORYLATION and MYC_TARGETS_V1 pathways, which are closely associated with increased metabolic activity and proliferative capacity in PDAC cells, recognized as key drivers of carcinogenesis and progression [24]. Additionally, ductal subclusters were enriched in APOPTOSIS, TNFA_SIGNALING_VIA_NFKB, and MTORC1_SIGNALING, suggesting mechanisms for stress adaptation and survival underlying the aggressive phenotype of PDAC [25] (Figure 2C).

In malignant PRE subclusters, we observed distinct patterns among persistently dysregulated genes. The most aggressive ductal cell groups (C20, r = 0.94; C23, r = 0.91) displayed high expression of persistently upregulated genes, whereas the most aggressive acinar cell groups (C18, r = 0.83; C5, r = 0.20) exhibited high expression of persistently downregulated genes (Figure 2D). We investigated the clinical relevance of genes overlapping between trajectory-associated and persistently dysregulated sets by performing survival analysis in the TCGA PDAC cohort. Two representative overlapping genes, *IFI27* and NBEAL1, were significantly associated with poor patient prognosis (*IFI27*, *p* = 0.0116; NBEAL1, *p* = 0.0196), suggesting their potential roles in tumor progression (Figure 2E).

Collectively, our systematic single-cell analyses delineate and functionally characterize preneoplastic subclusters within ductal and acinar cells, elucidating their dynamic roles in PDAC carcinogenesis.

### 2.3. Macrophage-Driven Immune Microenvironment Remodeling During PDAC Carcinogenesis

**Figure 3:**
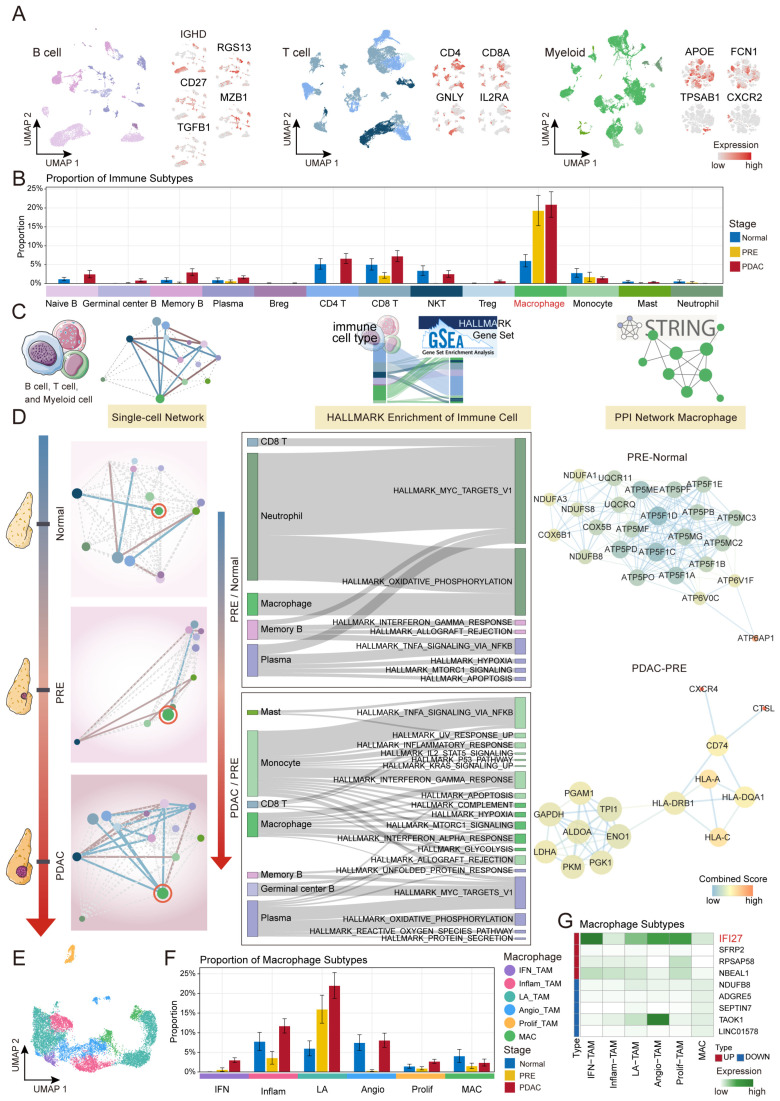
Immune cell landscape and functional dynamics across disease progression. (**A**) UMAP visualizations of B, T, and myeloid cell subclusters, colored by cell subtype as indicated. Adjacent panels show the expression of representative marker genes for each population. (**B**) Proportional distribution of immune cell subtypes across Normal, PRE, and PDAC stages. (**C**,**D**) Immune cell network and functional interaction analysis. (**C**) Schematic overview of the 3C analysis workflow. (**D**) Immune cell network construction and functional profiling. **Left**: Single-cell networks for Normal, PRE, and PDAC stages, with nodes representing cell types (colored and sized by identity and proportion) and edges indicating intercellular correlations. Edges represent Spearman correlations between cell-type centroids and are colored by sign and magnitude: positive correlations (ρ > 0.5) are shown in brown (reddish-brown), negative correlations (ρ < −0.5) in blue, and weak/non-significant correlations (|ρ| ≤ 0.5) as grey dotted lines. Macrophage nodes are highlighted with red circles. **Center**: Sankey plots illustrating hallmark pathways enriched for upregulated genes in immune cells (**top**: PRE vs. Normal; **bottom**: PDAC vs. PRE). **Right**: PPI networks of upregulated genes in macrophages (**top**: PRE vs. Normal; **bottom**: PDAC vs. PRE). (**E**) UMAP visualization of macrophage subclusters colored by subpopulation. **Right**: Dot plot depicting the expression of selected marker genes across macrophage subtypes. (**F**) Proportional distribution of macrophage subclusters across Normal, PRE, and PDAC stages. (**G**) Heatmaps showing persistently upregulated (**top**) and downregulated (**bottom**) genes across macrophage subtypes.

The initiation and progression of cancer are shaped not only by malignant cells but also by a dynamic and heterogeneous immune microenvironment [26]. To delineate immune cell remodeling across PDAC carcinogenesis, we comprehensively profiled immune cell composition and dynamics. Subclustering resolved principal immune subtypes, including B cells (naive, germinal center, memory, plasma, regulatory), T cells (CD4, CD8, NKT, regulatory), and myeloid cells (macrophages, monocytes, mast cells, neutrophils) (Figure 3A). Notably, macrophage abundance rose sharply from Normal through PRE to PDAC (0.6–19.3–20.9%) (Figure 3B), underscoring their central role in tumor evolution. Persistent accumulation of tumor-associated macrophages (TAMs) is associated with enhanced immunosuppression and increased tumor proliferation, invasion, and immune evasion [27].

To elucidate cellular interactions and functional remodeling during PDAC progression, we constructed immune cell interaction, HALLMARK pathway enrichment, and PPI networks across Normal, PRE, and PDAC stages (Figure 3C,D). Correlation analysis showed that macrophages shifted from a strong negative association with NKT cells in Normal (ρ = −0.74), to negative correlations with CD8 T cells (ρ = −0.71) and neutrophils (ρ = −0.55) in PRE, and to broad negative correlations with multiple lymphocyte subtypes in PDAC (ρ = −0.51 to −0.74), alongside a positive correlation with plasma cells (ρ = 0.66). HALLMARK pathway analysis (Supplementary Table S5) revealed that, compared to Normal, upregulated genes in PRE macrophages were enriched in oxidative phosphorylation, whereas those in PDAC, compared to PRE, were associated with canonical malignancy-related pathways, including glycolysis, hypoxia, apoptosis, interferon response, MYC targets, and mTORC1 signaling. STRING-based PPI analysis revealed a mitochondrial oxidative phosphorylation cluster in PRE macrophages, with upregulated genes such as *ATP6AP1*, *NDUFA3*, and *NDUFA1* distributed within the network. In PDAC, central hub genes identified included *CXCR4* [28] and *CTSL* [29], while *HLA-A* and *HLA-C* exhibited high connectivity and formed a tightly interconnected module with other antigen presentation-related genes. Together, these findings demonstrate that macrophages orchestrate the formation of an immunosuppressive microenvironment and promote immune evasion during tumor progression [30].

To further elucidate the dynamic changes in macrophages during the carcinogenesis of PDAC, we annotated five tumor-associated macrophage subclusters (Prolif-TAM, Angio-TAM, LA-TAM, Inflam-TAM, IFN-TAM) and one healthy macrophage subpopulation (MAC) based on established markers [31] (Figure 3E). Analysis of the proportions of these macrophage subtypes across different stages revealed a sustained increase in LA-TAM and IFN-TAM populations during tumor progression, with LA-TAM showing the most pronounced expansion (Figure 3F). Further analysis of persistently dysregulated genes revealed that *IFI27*, characterized by sustained upregulation, was abundantly expressed in IFN-TAM, Angio-TAM, Prolif-TAM, and LA-TAM. In contrast, *TAOK1*, despite its overall persistent downregulation, demonstrated notably increased expression specifically within the Angio-TAM subset (Figure 3G).

Altogether, these findings highlight the dynamic changes in the immune microenvironment and the multifaceted reprogramming of macrophages during PDAC carcinogenesis.

### 2.4. Dynamic Intensification of Macrophage-Ductal and Acinar Crosstalk During PDAC Carcinogenesis

**Figure 4:**
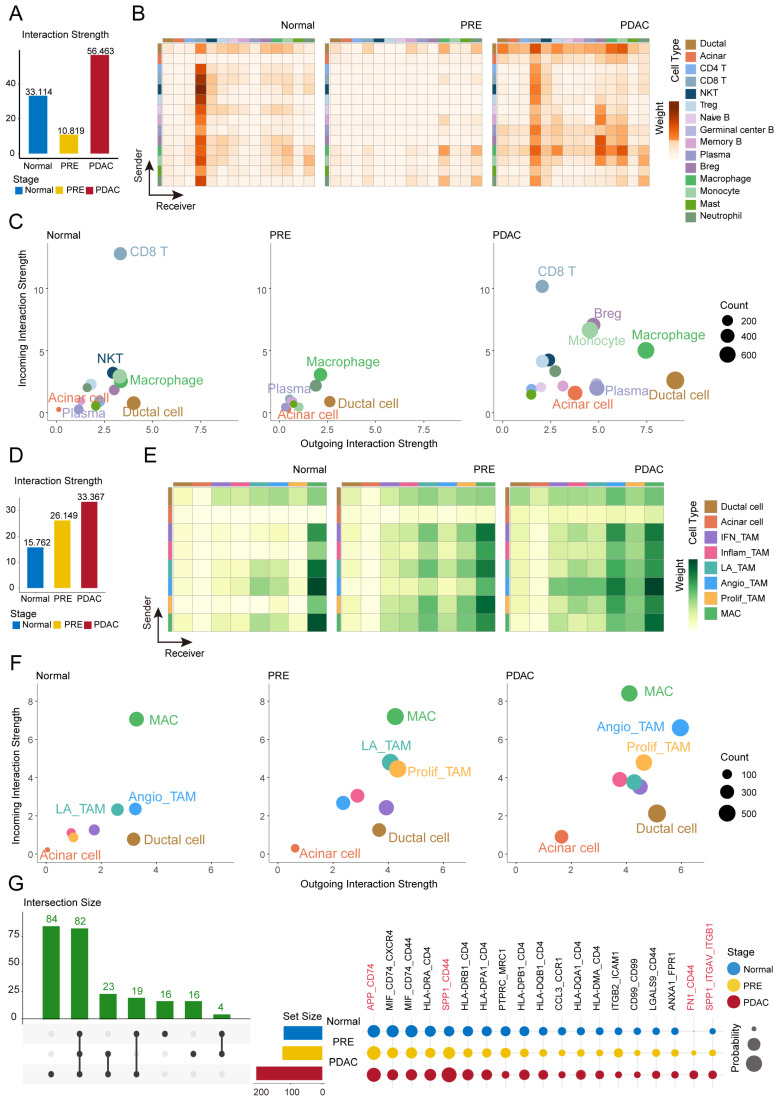
Cell–cell communication dynamics among immune, ductal, acinar, and macrophage subclusters across pancreatic carcinogenesis. (**A**–**C**) Analysis of interaction strength and signaling dynamics between immune cells and potential malignant ductal and acinar cells across Normal, PRE, and PDAC stages. (**A**) Interaction strength between immune cells and potential malignant ductal and acinar cells. (**B**) Heatmap illustrating stage-specific differences in interaction strength. (**C**) Scatter plots of incoming and outgoing signaling roles for each cell type; dot size represents interaction strength and color denotes cell identity. Axes and color mapping are consistent across stages. (**D**–**F**) Corresponding analyses for macrophage subclusters and potential pre-malignant ductal and acinar clusters. (**D**) Interaction strength between macrophage subclusters and potential pre-malignant ductal and acinar clusters across Normal, PRE, and PDAC stages. (**E**) Heatmap illustrating differential interaction strength among these stages. (**F**) Scatter plots depicting signaling roles of each cell population; graphical parameters as in (**C**). (**G**) UpSet plot (**left**) summarizing the shared and unique ligand–receptor pairs identified across Normal, PRE, and PDAC stages. Linking lines connect the set-membership dots and indicate which stages are included in each intersection. Dot plot (**right**) displaying the top 80 ligand–receptor pairs ranked by significance and interaction probability in each stage; dot size reflects interaction probability, and color indicates the stage.

Intercellular signaling is pivotal to carcinogenesis and microenvironmental reprogramming. To delineate these processes, we utilized CellChat [32] to systematically characterize cellular interactions across Normal, PRE, and PDAC stages. At each stage, communication networks were constructed for immune cells and malignant PRE clusters of ductal and acinar origin, as defined in the Methods. Our analysis revealed pronounced shifts in communication intensity during disease progression (Figure 4A): interaction strength was active in the Normal stage (33.114), declined substantially in PRE (10.819), and escalated in PDAC (56.463), reflecting extensive microenvironmental remodeling [33]. CD8 T cells were the principal signal recipients in the Normal stage. In PRE, despite the overall decline, macrophages continued to interact with malignant PRE ductal cells, highlighting their regulatory role. In PDAC, macrophage-driven communication increased, yielding a more dynamic signaling landscape (Figure 4B,C).

Analysis of macrophage subtypes revealed a progressive increase in signaling with malignant PRE cells across stages (15.762, 26.149, and 33.367 for Normal, PRE, and PDAC, respectively; Figure 4D). Communication between malignant PRE ductal cells and multiple macrophage subtypes was consistently elevated, especially among Angio-TAM, Prolif-TAM, and LA-TAM subtypes with high *IFI27* expression. While interactions between malignant PRE acinar cells and macrophages also intensified, these were less pronounced than those involving ductal cells (Figure 4E,F). Focusing on the most active ligand–receptor pairs between macrophage subtypes and malignant PRE cells at each stage (Figure 4G), we found consistently elevated *APP-CD74* signaling, suggesting a fundamental role in maintaining microenvironmental homeostasis [34]. In parallel, interaction probabilities for *SPP1-CD44* [35], *SPP1-ITGAV/ITGB1* [36], and *FN1-CD44* increased progressively from the Normal to PRE and PDAC stages, implicating these pathways in matrix remodeling and malignant progression.

Taken together, these results demonstrate that macrophage–ductal and acinar cell communication is initiated early and progressively intensifies from the PRE stage onward. This sustained remodeling of immune–ductal and acinar cell interactions characterizes the evolving PDAC microenvironment.

### 2.5. Consensus Subtypes and Drug Reversal Profiling Inform Precision Strategies in Pancreatic Carcinogenesis

**Figure 5:**
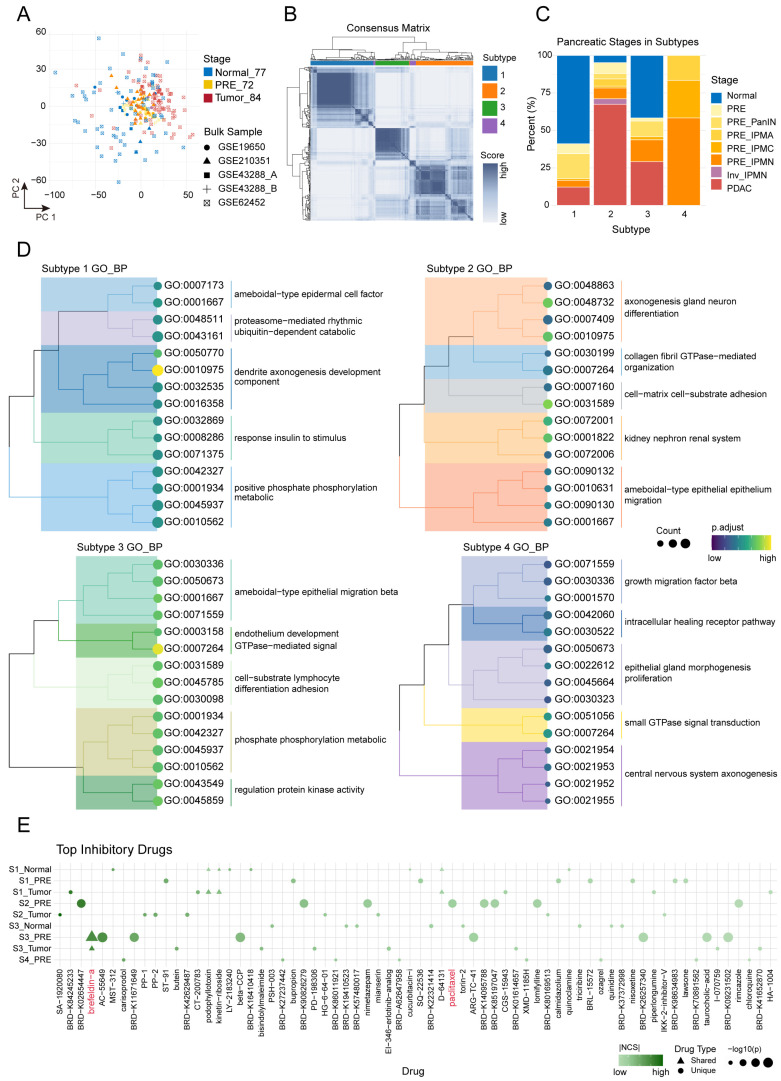
Molecular subtyping of pancreatic normal, preneoplastic, and tumor samples and identification of candidate therapeutic compounds. (**A**) PCA plot of batch-corrected bulk transcriptomic samples. Each point represents a sample, colored by stage (Normal, PRE, PDAC) and shaped by dataset of origin. (**B**) Consensus clustering heatmap of bulk samples based on ligand–receptor gene expression, identifying four molecular subtypes; color bars indicate cluster assignment. The list of ligand–receptor pairs used for clustering is provided in Supplementary Table S6. The diagonal blocks correspond to consensus clusters (molecular subtypes); each block groups samples that consistently co-cluster across resampling, with color intensity reflecting the consensus index (darker = higher co-clustering frequency). Sample subtype assignments are shown by the color bar. (**C**) Distribution of sample stages within each consensus cluster. (**D**) Tree plots depicting the top 15 enriched GO biological processes (adjusted *p* < 0.05) for each consensus subtype. Lines connecting nodes in the GO treeplots denote pairwise semantic similarity between GO terms as computed by pairwise_termsim; line connectivity indicates term relatedness and grouping. (**E**) Bubble plots of candidate compounds predicted by LINCS reversal analysis across consensus clusters and sample stages. Each point represents a LINCS perturbagen. The corresponding information is in Supplementary Table S8; bubble size reflects statistical significance (−log10P), color indicates absolute NCS, and shape denotes stage specificity.

To identify novel therapeutic strategies capable of intervening in pancreatic carcinogenesis, we comprehensively integrated bulk transcriptomic data from 77 Normal, 72 PRE, and 84 PDAC samples (Supplementary Table S1), applying rigorous normalization to eliminate batch effects (Figure 5A). Using ligand–receptor genes (Supplementary Table S6) that indicate high communication activity between macrophages and malignant preneoplastic ductal/acinar cells at the single-cell level, we performed consensus clustering of bulk samples (Figure 5B), ultimately delineating four molecular subtypes (S1–S4) with distinct distribution patterns (Figure 5C): S1(Subtype 1) was predominantly composed of Normal samples; S2 was enriched in PDAC samples; S3 exhibited a balanced distribution among the three stages; S4 consisted mainly of PRE samples.

Gene Ontology biological process (BP) enrichment analysis of differentially expressed genes among these subtypes (Figure 5D; Supplementary Table S7) revealed substantial functional heterogeneity. S1 (“homeostatic subtype”) was enriched for neurodevelopment, neurite morphogenesis, cytoskeletal remodeling, and protein phosphorylation regulation, reflecting maintenance of tissue architecture and signaling stability. S2 (“invasive subtype”) showed significant enrichment in cell adhesion, epithelial migration, gland development, and wound healing, indicating enhanced cellular motility and tissue remodeling underlying tumor invasion and microenvironmental adaptation. S3 (“transitional subtype”) encompassed diverse signaling pathways related to cell adhesion, migration, kinase activity regulation, and differentiation, suggesting dynamic functional regulation and a bridging role in tissue transformation and tumor progression. S4 (“preneoplastic subtype”) was associated with small GTPase signaling, neurodevelopment, gland development, and negative regulation of cell motility, implicating early microenvironmental remodeling and aberrant cell fate transitions that precede malignant progression.

Leveraging the subtype-specific molecular features, we systematically applied the LINCS drug perturbation reversal framework to screen candidate compounds with significant reversal potential for distinct molecular subtypes and stages (Figure 5E; Supplementary Table S8). After comprehensive screening of candidate drugs, we found that paclitaxel, identified for S2-PRE (preneoplastic samples from the S2 subtype), is the standard first-line chemotherapeutic for PDAC (e.g., nab-paclitaxel plus gemcitabine, proven to extend patient survival [37]), further supporting the scientific robustness and clinical significance of our methodology. Interestingly, brefeldin A (BFA), a molecule widely recognized in drug development but not yet used in clinical interventions at the preneoplastic stage, demonstrated a significant reversal effect in S3-PRE samples. Mechanistically, BFA acts by specifically inhibiting Arf1 GTPase, effectively blocking protein transport from the endoplasmic reticulum to the Golgi apparatus, thereby inducing endoplasmic reticulum stress and ultimately leading to apoptosis.

Specifically, BFA halts protein transport from the endoplasmic reticulum to the Golgi apparatus [38]. This process leads to the abnormal accumulation of secretory proteins within the endoplasmic reticulum, triggering endoplasmic reticulum stress. Prolonged cellular stress activates Golgi autophagy as an adaptive mechanism and, over time, induces genomic instability, such as aneuploidy, or directly initiates apoptotic pathways, ultimately resulting in cell death [39,40]. Previous studies also show that BFA can reverse certain drug accumulation/extrusion defects in pancreatic tumor cells [41], enhance the efficacy of gemcitabine in gemcitabine-resistant cells [42], and trigger p53-independent tumor-cell apoptosis [43,44]. Given that approximately 50% of PDACs harbor TP53 mutations, this p53-independent pro-apoptotic activity is particularly relevant. In recent years, the anti-tumor potential of BFA has garnered increasing attention. For example, its ester derivatives and nitric oxide-releasing analogs have demonstrated excellent anticancer activity [45,46]; related research is also expanding its application as a natural product anticancer agent [47]. Our identification of BFA in this context highlights both its translational promise and the innovative nature of our screening strategy.

Overall, our integrative multi-omics analysis and drug reversal screening have delineated the key molecular heterogeneity of pancreatic cancer, providing novel avenues for the development of subtype-specific therapeutic strategies.

## 3. Discussion

Early diagnosis and surgical resection represent the best options for long-term survival in patients with PDAC [2]. The precursor lesions that give rise to PDAC represent a critical window for intervention in carcinogenesis. Although recent advances have improved the understanding of PDAC mechanisms, the dynamic progression and cellular heterogeneity of the disease remain incompletely characterized [3]. In this study, we integrated single-cell and bulk transcriptomic data from normal pancreas, precursor lesions, and tumors to construct a comprehensive molecular landscape of PDAC carcinogenesis, with particular emphasis on identifying therapeutic targets for early intervention.

Single-cell resolution enables precise delineation of cellular mechanisms underlying carcinogenesis [9]. Changes in cell ratios revealed a negative correlation between immune cell numbers and epithelial cells (ductal and acinar cells), with acinar-to-ductal metaplasia (ADM) occurring significantly in the PRE stage. Given that the origin of PDAC cells is still inconclusive [12,13], both ductal and acinar cells are regarded as key candidates for malignant transformation in this study. By comparing PRE subclusters with malignant cells identified by inferCNV, we pinpointed PRE subclusters with malignant-like copy-number profiles. Combined with trajectory and functional enrichment analysis, we validated the transformative potential of these premalignant subclusters. Additionally, a set of persistently aberrant genes was identified throughout carcinogenesis, showing close associations with malignant transformation and poor prognosis, underscoring their critical role in early PDAC progression.

The occurrence of pancreatic cancer is closely related to the remodeling of the immune microenvironment [26]. In our study, macrophage proportions increased progressively from Normal through PRE lesions to PDAC, and higher macrophage abundance correlated with signatures of immunosuppression, tumor proliferation, and invasion [27]. While PRE macrophages primarily function in oxidative phosphorylation, PDAC stages activate glycolysis, hypoxia, and immune escape pathways, with *CXCR4* [28] and *CTSL* [29] acting as key hubs. TAM subtypes (such as LA-TAM and IFN-TAM) continue to expand during tumor progression, further highlighting the central role of macrophages in microenvironment remodeling [30].

Cell interaction network analysis reveals that microenvironmental signaling is dramatically reshaped during disease progression [33]. CellChat analysis shows that interactions between macrophages and malignant ductal cells remain active in PRE. Further, in PDAC, macrophages dominate signaling. Signaling between macrophage subtypes and malignant ductal cells continues to increase with disease progression, with ligand–receptor signals such as *APP-CD74*, *SPP1-CD44*, and *FN1-CD44* being highly expressed at all stages, highlighting the ongoing interaction between macrophages cells and ductal/acinar cells and their importance in microenvironmental evolution [34,35,36].

Based on highly active ligand–receptor gene pairs in macrophages and ductal/acinar cells, combined with bulk transcriptome samples from Normal, PRE, and PDAC stages, four molecular subtypes (S1–S4) were identified. LINCS drug reversal analysis was used to screen candidate drug compounds for each molecular subtype and stage. Paclitaxel was shown to be suitable for the S2-PRE subtype and serves as a first-line treatment for PDAC [37]. Brefeldin A demonstrated promising reversal potential in the S3-PRE preneoplastic stage, providing new avenues for early intervention [45,46,47].

## 4. Materials and Methods

### 4.1. Sample Collection

All single-cell and bulk transcriptomic datasets analyzed in this study were obtained from public repositories (GEO and GSA) and consisted entirely of human samples. The single-cell dataset included 22 normal pancreatic tissue (Normal), 31 preneoplastic lesions (PRE), and 39 pancreatic ductal adenocarcinoma (PDAC) samples. Notably, two of the PDAC samples were liver metastases, while the others were primary samples. The bulk transcriptomic dataset consisted of 77 Normal, 72 PRE, and 84 PDAC samples. Detailed information on dataset accession numbers, sample types, and stages is provided in Supplementary Table S1.

### 4.2. Single-Cell RNA-Seq Data Quality Control and Analysis

A total of 92 samples comprising 10 scRNA-seq datasets were included in this study. Cell quality control criteria included (1) the number of detected genes ranging from 200 to 5000; and (2) the proportion of mitochondrial genes less than 10% [10]. After quality control, 170,159 high-quality cells were retained for downstream analyses. Data integration was performed using Seurat (v5.3.0) [48]. Gene expression matrices were normalized using the LogNormalize method, and the top 2000 highly variable genes were identified using the vst method [49]. Batch effects were corrected using the Harmony algorithm (v1.2.3) [50], and the batch-corrected expression matrix was used for further analyses.

### 4.3. Clustering and Cell Type Annotation

Neighbor graphs were constructed using the first 30 harmony principal components (PC), and clustering was performed with the Louvain algorithm implemented in Seurat (v5.3.0) [48]. Clustering resolutions from 0.1 to 1.5 were tested, and a resolution of 0.9 was ultimately selected for downstream analyses. UMAP dimensionality reduction and visualization were then performed using the top 30 principal components to display the cellular clustering structure. Differential expression analysis for each cluster was conducted using the Wilcoxon rank-sum test, with thresholds of logFC > 0.5, min.pct = 0.5, and adjusted *p* value < 0.05. Cluster annotation was based on known marker genes and differential expression profiles. Subsequently, cells of each major lineage were extracted from the integrated dataset and subjected to the same clustering and annotation strategy for refined subpopulation delineation.

### 4.4. Identification of Malignant Cells and Malignant Preneoplastic Clusters

Copy number variations (CNVs) were inferred from single-cell RNA-sequencing data using inferCNV (v1.18.1) [51] to identify malignant cells at the PDAC stage. Raw count matrices were extracted from Seurat objects, using B cells as the reference population. The gene ordering file was constructed based on the GRCh38 genome. During analysis, the minimum average expression threshold was set to 0.1, and the denoising function was enabled. Cells from the PDAC stage exhibiting extensive CNV alterations relative to B cells were classified as malignant.

To further refine the identification of malignant PRE subclusters, dimensionality reduction and unsupervised clustering were performed separately on ductal and acinar cells. Subclusters with a composition of more than 50% preneoplastic-stage cells were defined as preneoplastic clusters. For each preneoplastic cluster, the Pearson correlation coefficient was calculated between its expression profile and that of PDAC-stage malignant cells identified by inferCNV. Clusters with a correlation coefficient exceeding 0.75 were designated as malignant PRE clusters. Notably, malignant PRE clusters may contain a minority of normal or tumor-stage cells.

### 4.5. Pseudotime Trajectory Inference

Pseudotime trajectory analysis was performed using Monocle2 (v2.14.0) [52,53,54] on the integrated Seurat object to investigate cellular differentiation processes. PRE cells, malignant PRE cells as defined in this study, and PDAC-stage cells were imported into Monocle2. Genes expressed in at least 10 cells with an average expression level of ≥0.1 were selected as the feature set for analysis. Differentially expressed genes (DEGs) were identified based on Seurat cluster information (seurat_clusters), and those with a q-value < 0.01 were used as ordering genes. Dimensionality reduction was performed using the DDRTree algorithm, and cells were ordered with default Monocle2 parameters to reconstruct developmental trajectories and infer lineage relationships. Pseudotime heatmaps were visualized in Origin version 2024 (https://www.originlab.com/) using results exported from R (version 4.3.3).

### 4.6. Construction of Stage-Specific Immune Networks and Correlation Analysis

To investigate dynamic interactions among immune cell subtypes during disease progression, stage-specific immune networks were constructed. The relative abundances of immune cell subtypes were quantified for each disease stage, and Spearman correlation coefficients were calculated to assess intercellular associations. Cell subtypes were represented as nodes, with centroid-based positioning; node color indicated cell type, node size reflected proportional abundance, and edge thickness represented the strength of correlation. Particular attention was given to shifts in the correlation patterns between macrophages and other immune populations across stages, providing insights into their roles in immune microenvironment remodeling and carcinogenesis.

### 4.7. Protein–Protein Interaction (PPI) Network Analysis

For each cell type, gene sets comprising differentially expressed genes identified between PRE and Normal (PRE-NOR group) and between PDAC and PRE (PDAC-PRE group) were separately submitted to the STRING database (https://string-db.org/) [55] with the organism set to Homo sapiens and a minimum required interaction score of 0.7 (highest confidence). Other parameters were set to default. The resulting PPI networks were exported in TSV format and analyzed in Cytoscape v3.10.2 (https://cytoscape.org/) [56]. Network topological properties, including degree, were calculated using the Network Analysis plugin to identify core proteins. Isolated nodes were excluded from further analysis.

### 4.8. Gene Set Enrichment and Visualization

HALLMARK pathway enrichment analysis was performed for cell clusters using the ‘clusterProfiler’ R package [57], with HALLMARK gene sets (“H” category) obtained from the MSigDB database via the ‘msigdbr’ package [58]. Enrichment was conducted using the ‘enricher’ function and visualized as a dot plot. In Result 3, significant HALLMARK pathways (*p*.adjust < 0.05) were aggregated across cell types.

Gene Ontology (GO) enrichment analysis was performed on subtype-specific differentially expressed genes using the ‘enrichGO’ function in ‘clusterProfiler’ [57], with multiple testing correction (Benjamini–Hochberg) and a q-value cutoff of 0.05.

### 4.9. Cellchat Analysis

Ligand–receptor interactions were inferred using the CellChat R package (v1.5.0) [32] based on single-cell transcriptomic data. Separate CellChat objects were generated for Normal, PRE, and PDAC stages, and intercellular communication networks were constructed using the integrated database. The ‘compareInteractions’ and ‘netAnalysis_signalingRole_scatter’ functions were applied to assess dynamic changes in communication strength and signaling roles across stages. Shared and stage-specific LR pairs were visualized using the ‘upset’ function. For each stage, the top 80 most active LR pairs were displayed as dot plots generated with ‘ggplot2’, with dot size representing interaction strength.

### 4.10. Consensus Clustering

We integrated samples from the Normal, PRE, and PDAC stages and identified ligand–receptor (LR) gene pairs exhibiting strong communication activity between macrophages and malignant PRE ductal/acinar cells. After identifying LR genes shared across all samples, we extracted their expression profiles from the normalized bulk transcriptome matrix. Using ConsensusClusterPlus (v1.73.0), we performed consensus clustering based on hierarchical clustering (hc) and Pearson correlation. The analysis involved 450 resamplings and 80% sample subsampling per iteration. We determined the optimal number of clusters (k = 4) by evaluating consensus matrices and cluster stability, and assigned molecular subtypes accordingly.

### 4.11. Differential Expression Analysis

We used the limma package (v3.65.1) to identify differentially expressed genes for each molecular subtype compared to all others (one-vs-rest). We constructed the design matrix according to subtype classification and applied linear modeling with empirical Bayes moderation to the normalized expression data. We defined significant genes as those with a Benjamini–Hochberg adjusted *p*-value < 0.05 and an absolute log2 fold change (|logFC|) > 0.5.

### 4.12. Drug Reversal Analysis

Drug-repositioning candidates were identified by matching group-specific transcriptional signatures to the LINCS L1000 database [59] (snapshot downloaded 25 June 2025). For each subtype stage, we performed differential expression on batch-corrected data (expr_combat) using limma and selected genes with Benjamini–Hochberg adjusted *p*-value < 0.05 and |log2 fold change| > 0.15. The top 200 up- and down-regulated genes per group were mapped to Entrez IDs; query signatures with fewer than five Entrez IDs were excluded (min.genes = 5). Query signatures were constructed with qSig and matched to the LINCS reference; for each perturbagen, we report the mean normalized connectivity score (mean NCS; column ‘similarity’ in Supplementary Table S8) and the minimum permutation-based WTCS *p*-value (*p*.value). Only perturbagens with mean NCS < 0 were retained and ranked by permutation-based WTCS *p*-value (*p*.value).

### 4.13. Statistical Analysis

All graphical constructions in this study were performed using R software (version 4.3.3). To determine statistical differences between groups, we used the Wilcox test to determine the statistical significance of differences. *p* < 0.05 was defined as statistically significant.

## Data Availability

The scRNA-seq data derived from public domain resources are available in Gene Expression Omnibus (GEO) and Genome Sequence Archive (GSA), including GSE205049, GSE197177, GSE155698, GSE212966, GSE165399, GSE229413, GSE254829, GSE263733, and CRA001160. The bulk transcriptomic data presented in this study are available in GEO, including GSE43288, GSE19650, GSE210351, and GSE62452.

## Supplementary Materials

The following supporting information can be downloaded at: https://www.mdpi.com/article/10.3390/ijms262412031/s1.
