# Supplementary material for: Elucidating Pancreatic Ductal Adenocarcinoma Carcinogenesis at Single-Cell Resolution and Identifying Subtype Specific Drug Candidates
Source: Int J Mol Sci. 2025 Dec 14;26(24):12031. doi: 10.3390/ijms262412031 (PMC12732769; doi:10.3390/ijms262412031)
Supplement: Supplementary file 1 [file ijms-26-12031-s001.zip › Supplementary Figures.pdf]

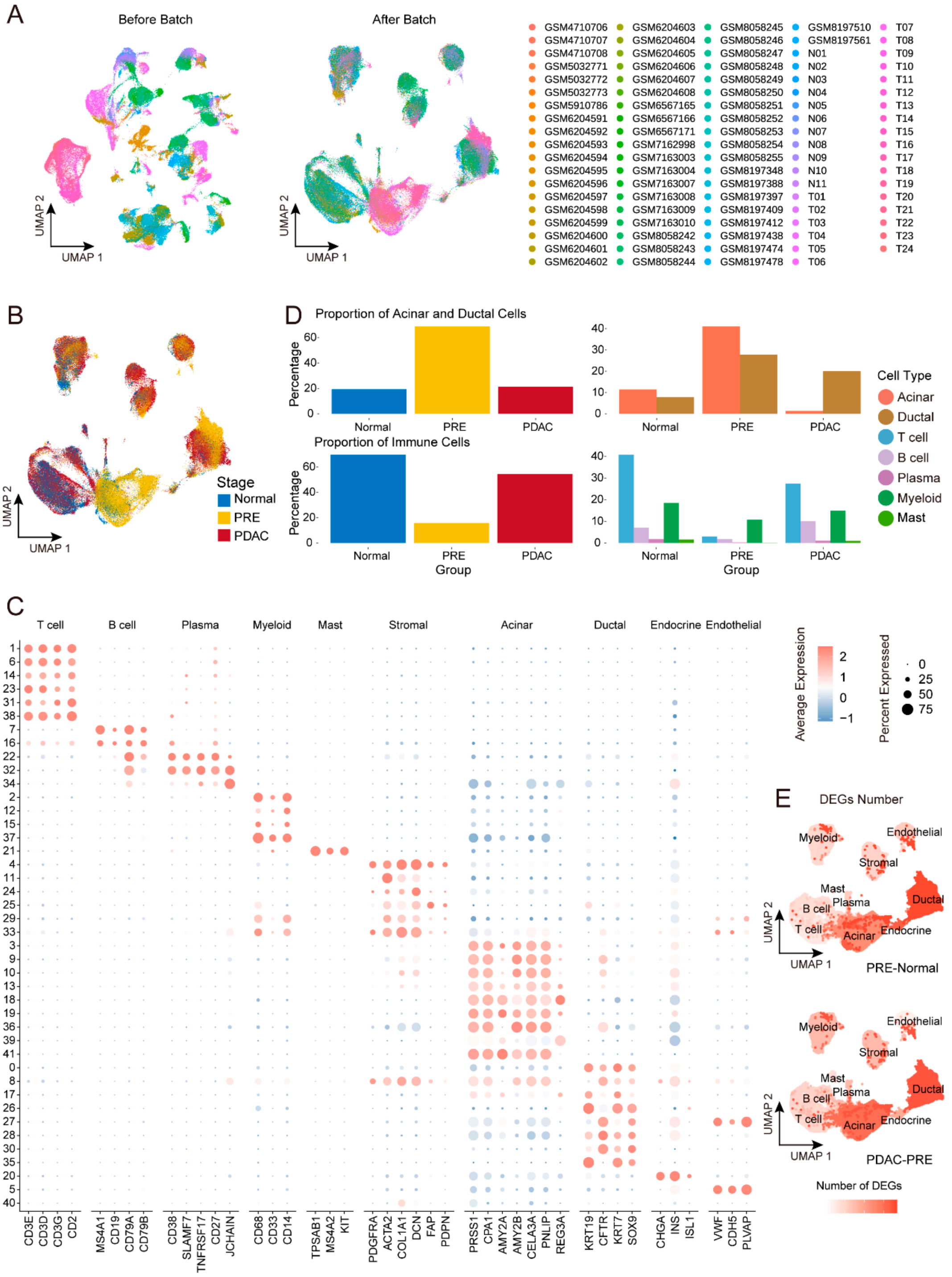

Supplementary Figure S1. Data integration, annotation, and cellular composition.

(A) UMAP visualization of samples before (left) and after (right) batch effect correction, colored by sample ID.

(B) UMAP visualization of 170,159 single cells colored by stage (Normal, PRE, PDAC).

(C) Dot plot of selected marker gene expression across cell clusters for cell type annotation.

(D) Bar plots showing the proportions of ductal and acinar cells (top) and immune cell subtypes (bottom) across Normal, PRE, and PDAC stages (left: overall; right: cell type-specific).

(E) Heatmap showing the number of DEGs per cell type for PRE versus Normal and PDAC versus PRE comparisons.

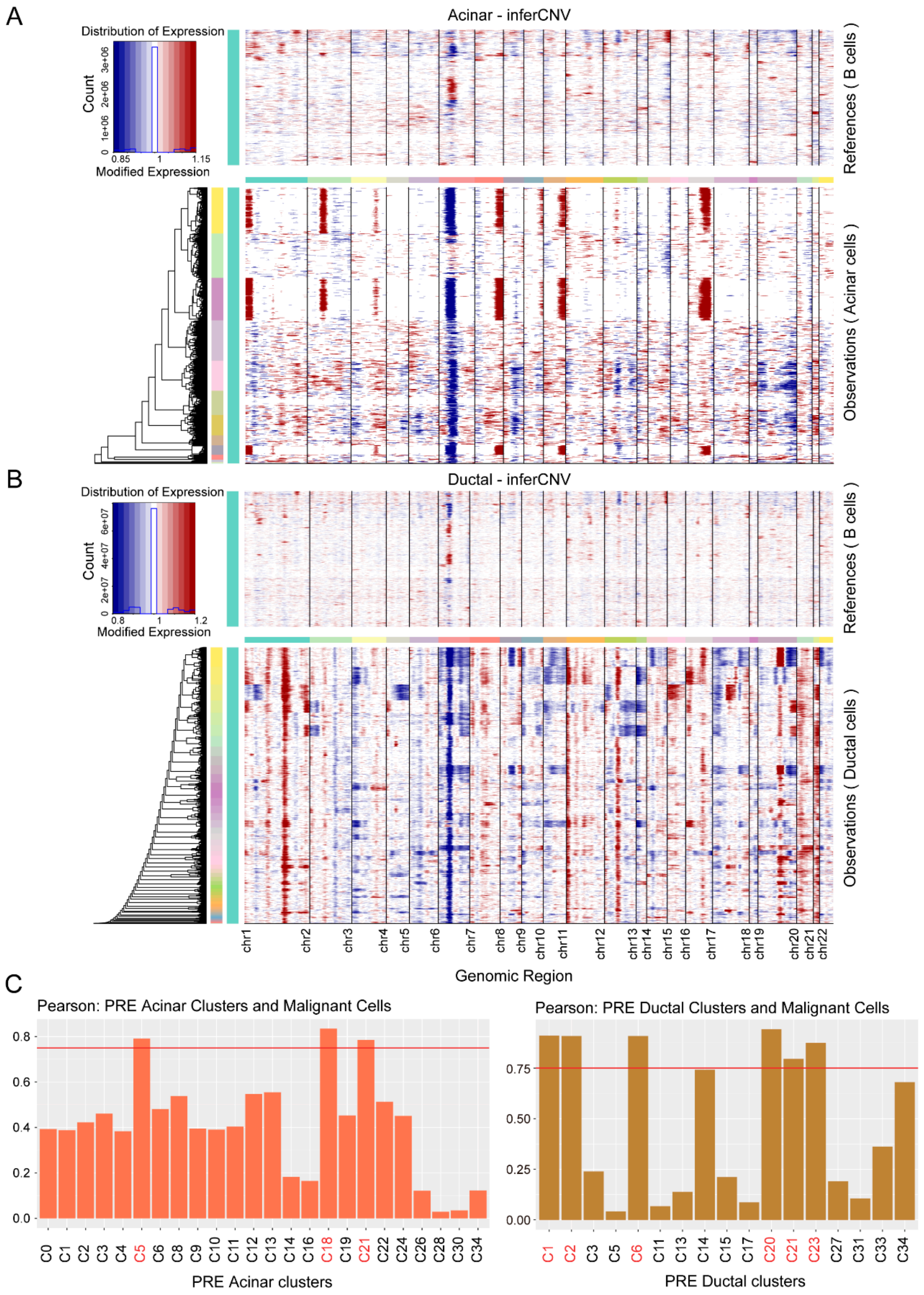

Supplementary Figure S2. Identification of malignant PRE clusters.

(A-B) InferCNV analysis of acinar (A) and ductal (B) cells in the PDAC stage, with B cells as reference.

(C) Pearson correlation between each PRE subcluster and PDAC-stage malignant cells identified by inferCNV. Left: acinar; right: ductal. A red line indicates a Pearson correlation coefficient of 0.75.
